# Flower-like ZnO-Ag_2_O composites: precipitation synthesis and photocatalytic activity

**DOI:** 10.1186/1556-276X-8-536

**Published:** 2013-12-19

**Authors:** Lingling Xu, Bo Wei, Weilong Liu, Hailin Zhang, Chunyan Su, Jixin Che

**Affiliations:** 1 Key Laboratory of Photonic and Electric Bandgap Materials, Ministry of Education, School of Physics and Electronic Engineering, Harbin Normal University, Harbin 150025, People’s Republic of China; 2 Department of Physics, Harbin Institute of Technology, Harbin 150080, People’s Republic of China; 3 Department of Physics, Harbin University, Harbin 150086, People’s Republic of China; 4 The Aviation University of Air Force, Changchun 130022, People’s Republic of China

**Keywords:** ZnO, Ag_2_O, Photocatalytic activity, Composite

## Abstract

Ag_2_O-decorated flower-like ZnO composites were fabricated through a chemical precipitation process. X-ray diffraction analysis confirms the co-existence of cubic Ag_2_O and wurtzite ZnO phases. Scanning electron microscopy images reveal Ag_2_O nanoparticles located on the rough surface of ZnO flowers. The photocatalytic activities of the composites with various mole ratios were evaluated by the degradation of methyl orange (MO) under ultraviolet irradiation, which confirms that the composite shows superior activity to that of pure ZnO and Ag_2_O. The improvement can be ascribed to the deposited Ag_2_O forming the p-n junction at the interface of ZnO and Ag_2_O, resulting in the transfer of photocarriers and suppressing the electron–hole recombination rate.

## Background

Semiconductor photocatalysts for clean hydrogen energy production and environment decontamination have attracted much interest [1,2]. When the excitation energy is higher than or equal to the band gap energy of the semiconductor, photoinduced electron–hole pairs would be generated and utilized in initiating oxidation and reduction of organic compounds. ZnO can be used as a photocatalyst and has drawn increasing attention because its photocatalytic activity is comparable to that of TiO_2_[3,4]. It has been reported that the photocatalytic activity is closely correlated with the morphology of photocatalysts [5]. Hierarchical ZnO with flower-like morphology shows promising application in decomposition of organic pollutant due to the increased optical absorption efficiency and large specific surface area [6,7]. However, due to the wide band gap of ZnO (3.2 eV), only a few part of natural solar radiation can be utilized and the photogenerated electron and hole pairs are liable to recombination, leading to low quantum yields. To improve its photocatalytic activity, one of strategy is to complex ZnO with a narrow-band semiconductor forming composites with a wider range light absorption and a reduced rate of the recombination of photogenerated electrons and holes.

Many reports focused on the enhanced photocatalytic performance of ZnO composites by coupling with suitable semiconductors, such as TiO_2_, ZnS, Bi_2_O_3_, and CuO [8-12]. The efficiency improvement on the degradation of organic dye can be ascribed to the effective separation of photoinduced carriers. Furthermore, the separation of photoinduced electrons and holes would be greatly enhanced and more efficient especially in the inner electric field, which was formed by a p-n-type semiconductor composite, such as CuO/ZnO and NiO/ZnO [12,13]. Ag_2_O is a p-type semiconductor with a band gap of about 1.3 eV. Recently, the modification of TiO_2_ and Bi_2_O_3_ was carried out using Ag_2_O nanoparticles decorated on the surface of photocatalysts [14-17]. Based on the heterojunction of Ag_2_O and TiO_2_, the recombination of photogenerated electrons and holes was greatly inhibited by transferring for the energy band matching and the build-up inner electric field, resulting in the photocatalytic activity enhancement [15,16]. However, to the best of our knowledge, there is no report in the literature on the photocatalytic properties of the p-n junctions of hierarchical mesoporous ZnO-Ag_2_O composites.

In this paper, flower-like ZnO-Ag_2_O composites were fabricated through a chemical co-precipitation process. The as-prepared composite including Ag_2_O particles deposited on the petal surfaces of ZnO flowers shows high crystallization. Compared with ZnO flowers and Ag_2_O particles, the photocatalyst ZnO-Ag_2_O composites with wide mole ratios exhibited enhanced photocatalytic properties that was confirmed by the degradation of methyl orange (MO) under ultraviolet irradiation.

## Methods

### Preparation of flower-like ZnO

All the chemicals used for the synthesis of flower-like ZnO are analytical grade reagents. Zinc nitrate solution (0.001 M) was prepared by dissolving a proper amount of Zn (NO_3_)_2_ in deionized water. The materials - 20 mL of Zn (NO_3_)_2_ solution, 20 mL of deionized water, 0.25 g of sucrose, and 1.2 g of urea - were added into a 50-mL Teflon-lined stainless steel autoclave. The autoclave was sealed, heated at 90°C for 2 h, and finally cooled to room temperature naturally. The white precipitation (precursor) was filtered and washed several times with deionized water, followed by drying in air at 90°C for 2 h. The precipitations were heat-treated at 600°C in air for 2 h (heating rate of 5°C min^−1^) in a muffle furnace to obtain the final hierarchical ZnO flowers.

### Preparation of Ag_2_O nanoparticles

Ag_2_O nanoparticles were synthesized from AgNO_3_, NaOH, and polyethylene glycol 8000 (PEG-8000) aqueous solution by the precipitation method. Firstly, 1.75 g of AgNO_3_ and 0.2 g of PEG-8000 were dissolved in 100 mL of deionized water. After a continuous stirring for 15 min, 0.05 M NaOH aqueous solutions were dropped into the above aqueous solution with the final pH = 14. Finally, Ag_2_O nanoparticles were washed thoroughly with deionized water followed by drying in air at 90°C for 2 h.

### Chemical synthesis of flower-like ZnO-Ag_2_O composites

Flower-like ZnO-Ag_2_O composites with different mole ratios were prepared by the chemical precipitation method. A typical experimental process for the composite with a mole ratio of 1:1 is given as follows: 0.4 g of flower-like ZnO was dispersed in 100 mL of deionized water, and 2 g of PEG-8000 was added into the mixture in order to immerse the ZnO thoroughly. Subsequently, 1.8 g of AgNO_3_ was added to the suspension, and the mixture was stirred magnetically for 30 min. Then 0.2 M of NaOH was dropped into the above mixture with the final pH value of 14. Finally, flower-like ZnO decorated by Ag_2_O nanoparticles was washed repeatedly with deionized water followed by a filtration and drying in air at 90°C for 2 h. In order to assess the relationship between the component and the photocatalytic activity of the composites, variable mole ratios of ZnO to Ag_2_O composites were prepared through a similar process.

### Characterizations and photocatalytic testing

X-ray diffraction (XRD) measurement was carried out using a Rigaku-D/max 2500 diffractometer (Rigaku, Shibuya-ku, Japan) with Cu-Kα radiation (*λ* = 0.15418 nm) for crystallization identification. The morphology, particle size, and chemical composition of the product were examined by scanning electron microscopy (SEM; Hitachi S-4800, Chiyoda-ku, Japan). X-ray photoelectron spectroscopy (XPS) experiments were performed with a Thermo Fisher K-Alpha X-ray photoelectron spectrometer (Thermo Fisher Scientific, Waltham, MA, USA) using Al Kα radiation (12 kV, 6 mA). The binding energies of elements were calibrated using C 1*s* (284.6 eV) as reference. Room-temperature ultraviolet–visible (UV–vis) absorption spectrum was recorded on a spectrophotometer (PerkinElmer Lambda-35, Waltham, MA, USA) in the wavelength range of 300 to 800 nm. The UV–vis diffuse reflectance spectra (DRS) were measured using a Shimadzu UV-2550 spectrophotometer (Shimadzu, Kyoto, Japan). Room-temperature photoluminescence (PL) spectra were collected with a laser micro-Raman (JY HR800, HORIBA, Kyoto, Japan).

MO was employed as a representative dye pollutant to evaluate the photocatalytic activity of ZnO-Ag_2_O composites. Next, 0.02 g of ZnO-Ag_2_O composites was suspended into 60-mL 2 × 10^−5^ M of MO aqueous solution and stirred for 30 min in a 200-mL beaker in the dark to reach an adsorption/desorption equilibrium for MO on the surface of ZnO-Ag_2_O composites. Then the mixture was irradiated by 16-W ultraviolet irradiation (Philips, Amsterdam, The Netherlands) at room temperature. After the reaction mixture was irradiated for a given time, the samples of 4 mL were withdrawn at each time and centrifuged for 20 min. The quantitative determination of MO was performed by measuring its absorption with a UV–vis spectrophotometer (PerkinElmer Lambda-35).

## Results and discussion

**Figure 1:**
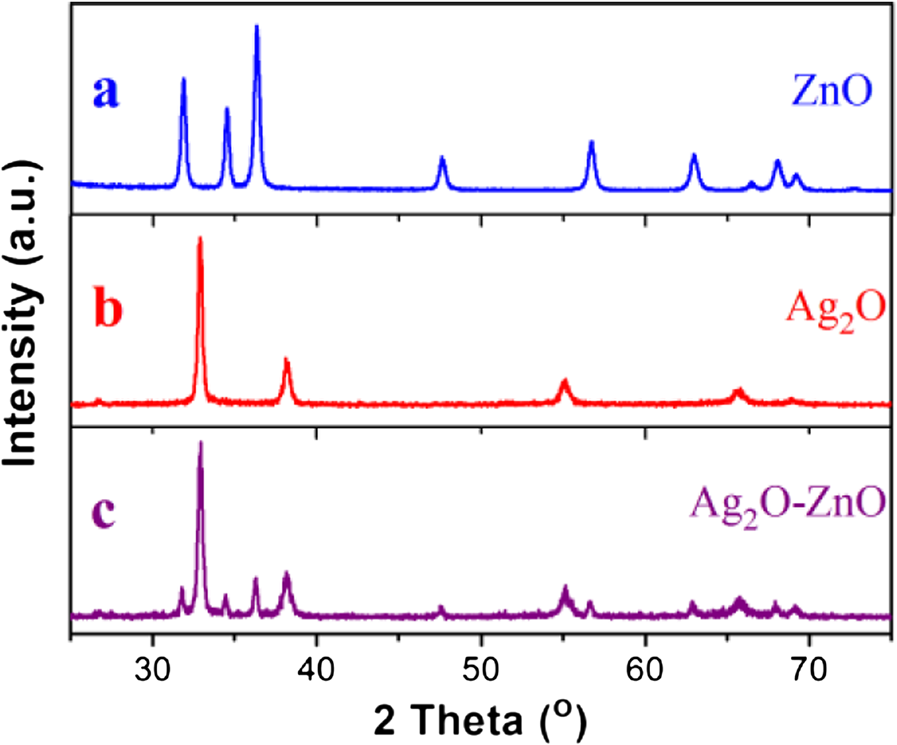
**XRD patterns of the as-synthesized products obtained. (a)** Pure ZnO, **(b)** pure Ag_2_O, and **(c)** ZnO-Ag_2_O composite.

The structure and phase purity of Ag_2_O nanoparticles, flower-like ZnO, and ZnO-Ag_2_O (1:1) composites were examined by XRD, and the patterns are shown in Figure 1. The diffraction peaks of the ZnO consist of three strong diffraction peaks, which can be mainly indexed as the wurtzite phase of ZnO (JCPDS card no. 36–1451) in Figure 1a. Meanwhile, the diffraction peaks in Figure 1b can be indexed to the cubic structure of pure Ag_2_O (JCPDS card no. 76–1393), with no additional peak detected, indicating the pure phase of Ag_2_O products. For the composite sample, the diffraction peaks in Figure 1c can be ascribed to two sets of strong diffraction peaks (JCPDS card nos. 36–1451 and 76–1393), revealing that ZnO and Ag_2_O coexist in the composite. The relative intensity of diffraction peaks in Figure 1c shows that the content of Ag_2_O is much more than that of ZnO for its intense and sharp diffraction peaks.

**Figure 2:**
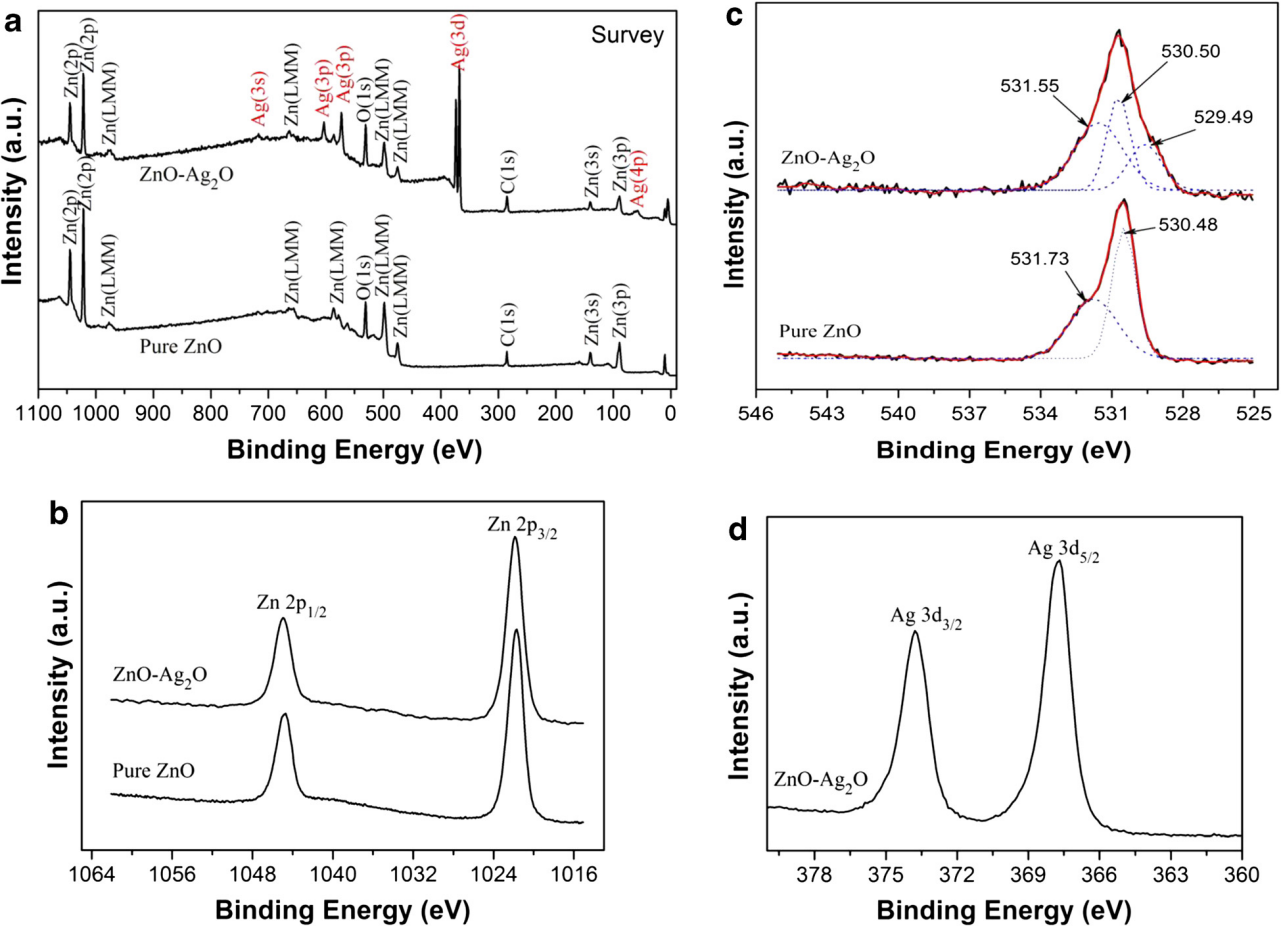
**XPS spectra of the ZnO-Ag**_**2**_**O composites and pure ZnO. (a)** Survey XPS spectrum, **(b)** Zn 2*p*, **(c)** O 1*s*, and **(d)** Ag 3*d*.

To investigate the surface compositions and chemical states of the as-prepared ZnO-Ag_2_O (1:1) composite, XPS was carried out, and the results are shown in Figure 2. The full-scan spectrum in Figure 2a shows the presence of C, O, Zn, Ag, and O peaks, which confirmed the presence of these elements in the products. The carbon peak comes from the adventitious carbon on the surface of the sample. The Zn 2*p* consists of two peaks positioned at 1,020.9 and 1,044.2 eV for Zn 2*p*_3/2_ and Zn 2*p*_1/2_ (Figure 2b), which were observed in both ZnO-Ag_2_O composites and pure ZnO [18]. As Figure 2c shows, O 1*s* can be deconvoluted by three nearly Gaussian curves in the ZnO-Ag_2_O composite, indicating that there are three different O species in the sample. The lowest binding energy component of 529.5 eV is attributed to O^2–^ ions surrounded by Ag atoms with their full complement of nearest-neighbor O^2–^ ions [19]. The middle binding energy component is usually attributed to chemically adsorbed oxygen on the surface of the catalysts [20]. The highest component is attributed to O^2–^ ions in ZnO [21]. However, O 1*s* only can be deconvoluted by two nearly Gaussian curves in pure ZnO. The binding energy components of 530.5 and 531.7 eV are attributed to chemically adsorbed oxygen and O^2–^ ions in ZnO, respectively. The peaks with binding energies of 367.8 and 373.8 eV correspond to Ag 3*d*_5/2_ and Ag 3*d*_3/2_, respectively, which is a characteristic of Ag^+^ in the Ag_2_O product in Figure 2d [21]. Consequently, the as-synthesized products could be determined as ZnO-Ag_2_O composites based on the results of XRD and XPS measurements.

**Figure 3:**
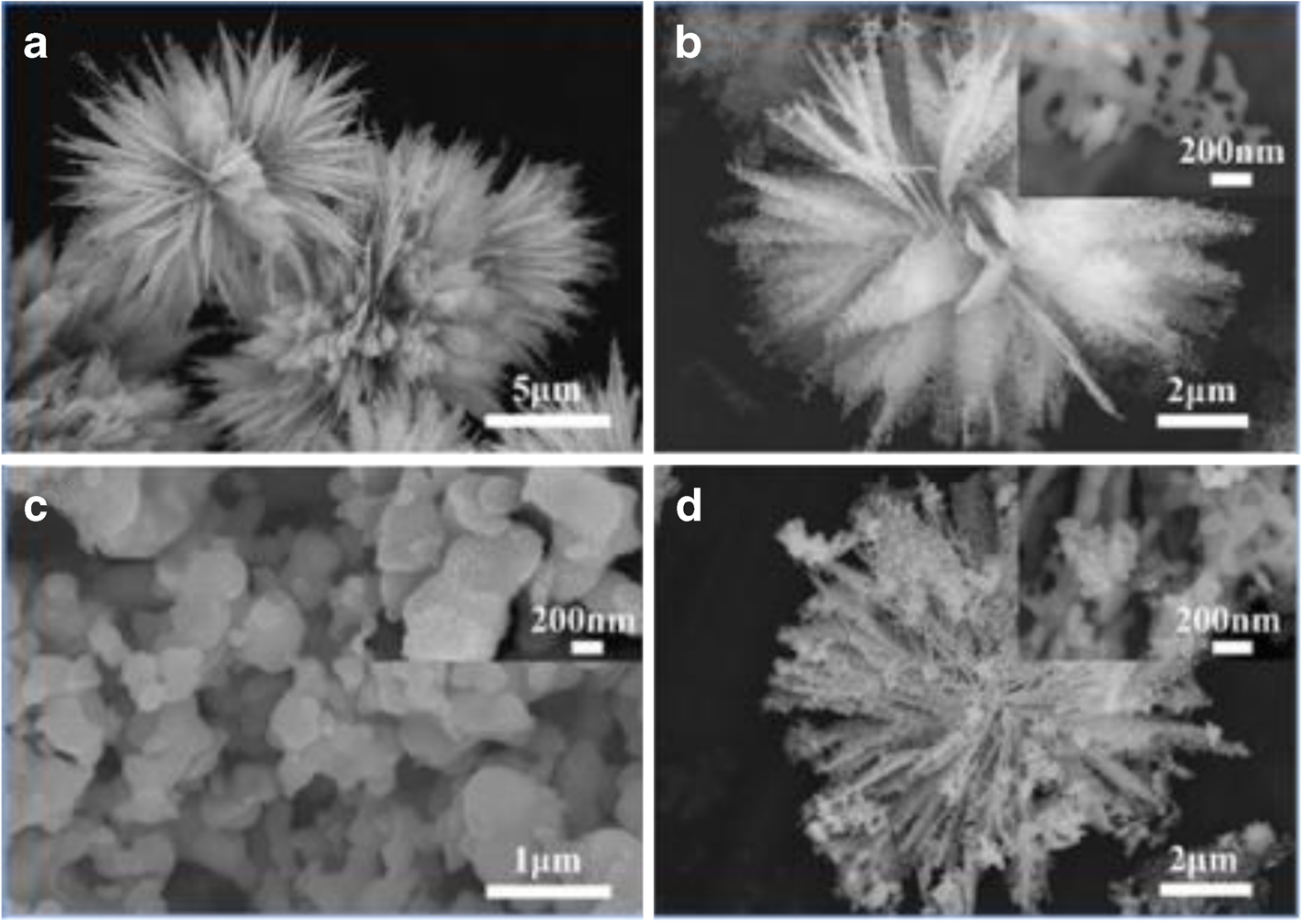
**SEM images of pure ZnO, pure Ag**_**2**_**O, and ZnO-Ag**_**2**_**O composite. (a)** Low-magnification SEM image of pure ZnO, **(b)** high-magnification SEM image of pure ZnO, and **(c, d)** typical images of pure Ag_2_O and ZnO-Ag_2_O composite.

In order to obtain the detailed information about the morphology of the synthesized Ag_2_O nanoparticles, SEM observation of flower-like ZnO and ZnO-Ag_2_O (1:1) composites was carried out, and the results are given in Figure 3. Figure 3a shows the SEM image of ZnO flower by the hydrothermal process. It reveals that the ZnO has a diameter of 5 to 10 μm and possesses a flower-like rough surface with petals emitted from the center. A typical ZnO flower image is shown in Figure 3b. Obviously, it is about 1 μm at the widest point of the flower petals which are ended with a tip. Moreover, there are a large amount of holes on the petals, which can greatly enlarge the contact area between the organic dyes and ZnO. The ending part of saw-like petals is shown as inserted in Figure 3b. It can be seen that holes on the petals present an irregular shape with an average diameter below 100 nm. Considering the annealing process, it can be deduced that the holes are coming from amounts of gases evaporating with the decomposition of the precursor at the relatively high temperature. The rough surfaces of ZnO provide a very good platform to locate Ag_2_O nanoparticles in high density during the co-precipitation process. Figure 3c shows the morphology of the Ag_2_O nanoparticles obtained by the precipitation method. Obviously, the diameter of Ag_2_O particles is in the range of 100 to 500 nm. The enlarged view as inserted in Figure 3c shows that the Ag_2_O presents a rough surface with small spherical particles. For the composited sample, the morphology maintained the flower of ZnO, while Ag_2_O clusters were observed on the petals. From the insert in Figure 3d, it shows that the Ag_2_O cluster was composed of dozens of Ag_2_O nanoparticles.

It is known that MO dyes are usually chosen as model pollutants to simulate the actual photocatalytic degradation of organic pollutants. The degradation efficiency was calculated using Equation 1:

(1) $$\mathrm{Degradation}\;\left(\%\right)=\frac{C=C_{0}-\Delta C}{C_{0}\times 100}=\frac{A=A_{0}-\Delta A}{A_{0}\times 100}$$

where *C*_0_ represents the initial concentration, Δ*C* represents the changed concentration, *C* represents the reaction concentration, *A*_0_ represents the initial absorbance, Δ*A* represents the changed absorbance, and *A* represents the reaction absorbance of the MO at the characteristic absorption wavelength of 464 nm.

**Figure 4:**
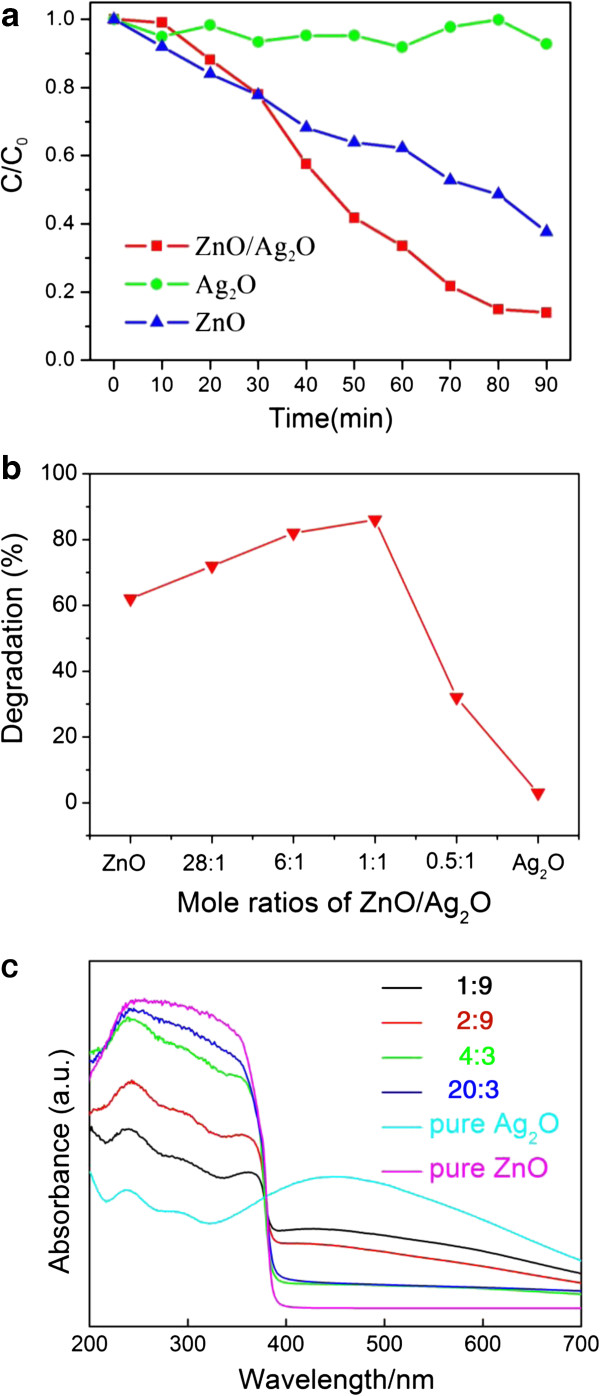
**Different experiments conducted to ZnO, Ag**_**2**_**O, and ZnO-Ag**_**2**_**O composites.** Photocatalytic degradation of MO in the presence of **(a)** pure ZnO, pure Ag_2_O, and ZnO-Ag_2_O composites under UV light irradiation; **(b)** different weight ratios of ZnO and Ag_2_O in 90 min; and **(c)** UV–vis diffuse reflectance spectra of pure Ag_2_O, ZnO, and Ag_2_O/ZnO composites with variable contents.

In the experiments, the photocatalytic activities of the as-prepared samples with different ZnO-Ag_2_O composites, pure ZnO flowers, and Ag_2_O particles are shown in Figure 4a. Surprisingly, the ZnO-Ag_2_O (1:1) composite exhibits superior photocatalytic activity, which is higher than that of pure ZnO flowers and Ag_2_O nanoparticles; for example, the required time for an entire decolorization of MO over ZnO-Ag_2_O catalysts is less than or equal to 90 min, much shorter than the corresponding value over pure ZnO flower and Ag_2_O particles. Moreover, the correlation between the photocatalytic activity and the component mole ratios is shown in Figure 4b. Obviously, the photocatalytic activity increases gradually with an increase of the Ag_2_O mole ratios (1:1 > 6:1 > 28:1 > 0.5:1) except ZnO-Ag_2_O (0.5:1). The composite ZnO-Ag_2_O (1:1) performed the highest activity among the samples we examined during a given time for 90 min. In addition, the ZnO-Ag_2_O composite shows higher photocatalytic activity than the pure components, ZnO and Ag_2_O. UV–vis diffuse reflectance spectra of pure Ag_2_O, ZnO, and Ag_2_O/ZnO composites with variable contents are shown in Figure 4c. Obviously, the absorption in the UV range is gradually quenched, while there is an obvious increase in the visible light range with the elevated loading of Ag_2_O. As for the UV light-excited photocatalytic process, the ability of UV light absorption is crucial for the effective excitation of photoinduced electron and holes. Thus, the photocatalytic activity would be determined by both the quantity of excited photoinduced carriers and the effective separation process in the inner electric field.

**Figure 5:**
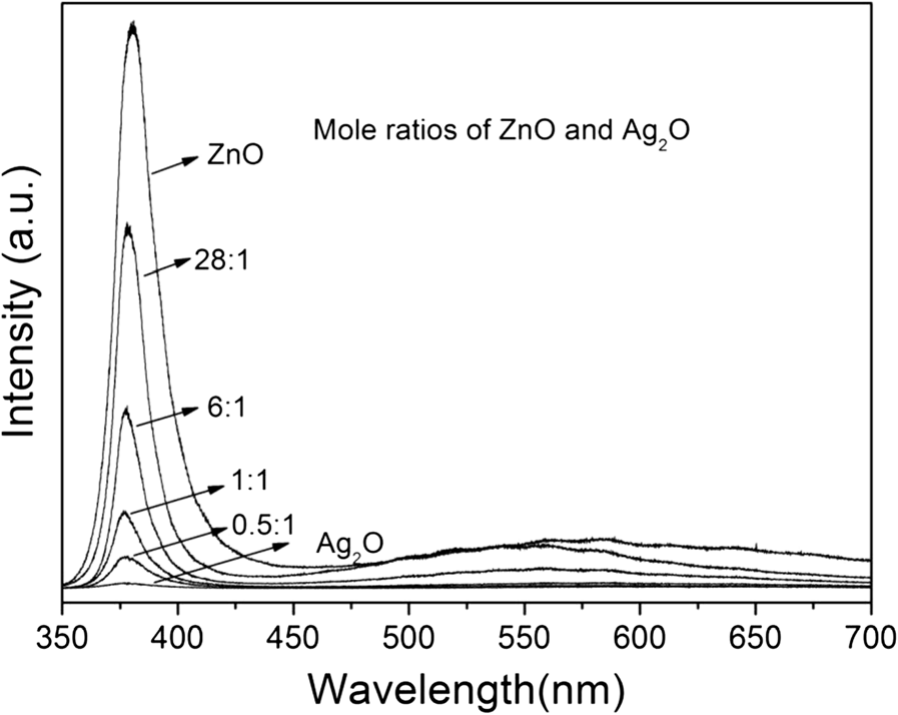
**PL spectra of pure ZnO, pure Ag**_**2**_**O, and ZnO-Ag**_**2**_**O composite at room temperature.**

Room-temperature photoluminescence measurements are widely used to characterize semiconductor nanoparticles, which possess a broad range of absorption, narrow emissions with high quantum yields, and size-tunable emission wavelength. The emission spectra of pure ZnO and ZnO-Ag_2_O composites excited at the emission peak of 325 nm are given in Figure 5. The photoluminescence spectrum of ZnO is composed of two emission bands: a near band edge emission positioned in the UV range and a visible emission band resulting from the defects [22,23]. Both the composite sample and pure ZnO present a band edge emission peak centered at 380 nm, while the band edge emission intensity of pure ZnO is drastically quenched by the increased loading of Ag_2_O particles, indicating the existence of a direct interaction between Ag_2_O and ZnO enhancing the nonirradiative relaxation of excitons formed in ZnO. The results demonstrate that the Ag_2_O particles block both direct and trap-related charge carrier recombination pathways since Ag_2_O particles on the ZnO surface can extract electrons from the conduction band of ZnO and act as a sink which can store and shuttle photogenerated electrons [14,15].

**Figure 6:**
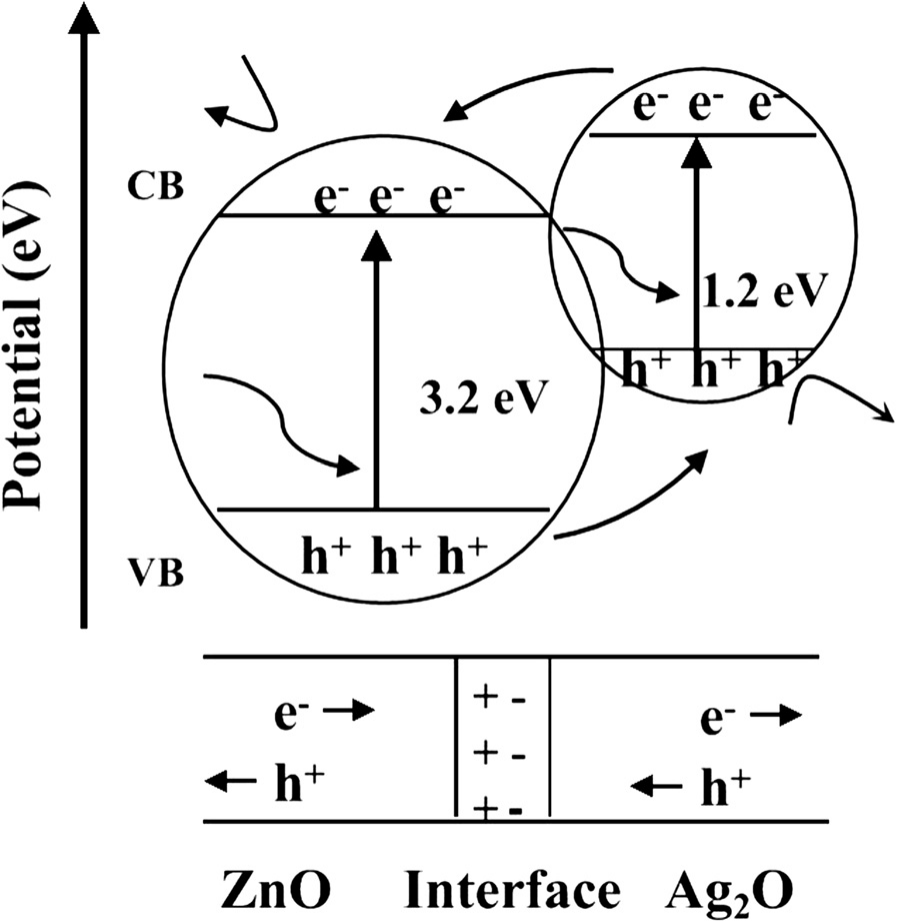
Schematic diagram of electron–hole separations at the interface and in both semiconductors.

As shown in Figure 6, the schematic band structure of the synthesized ZnO-Ag_2_O composite was proposed to discuss the possible process of the photocatalytic degradation of MO. When the catalysts are excited by ultraviolet light irradiation, electrons (e^−^) in the valence band (VB) can be excited to the conduction band (CB) with simultaneous generation of the same amount of holes (h^+^) in the VB, as demonstrated in Equations 2 and 3. The photoactivated electrons and holes in the ZnO-Ag_2_O composite could inject into a reaction medium and participate in the photocatalytic reaction process. The photoinduced holes (trapped by H_2_O) produce hydroxyl radical species (·OH) and the photoinduced electrons (trapped by O_2_ and H_2_O) produce hydroxyl radical species (·OH), which are extremely strong oxidants for the degradation of organic chemicals (Equations 4 and 5) [24]. It is known that ZnO is an n-type semiconductor while Ag_2_O is a p-type semiconductor. Thus, the Fermi levels of both n-type and p-type tend to obtain equilibrium, resulting in the energy bands of ZnO downward with the upward shifts of the Ag_2_O band. Moreover, there will be an inner electric field in the interface between ZnO and Ag_2_O in the composite, leading to a positive charge in the ZnO region and a negative charge in the Ag_2_O part. After the illumination of UV light, the photoinduced electrons and holes are created in the composite and subsequently transferred by the drive of inner field. Photoinduced electrons in the CB of Ag_2_O would move to the positively charged ZnO, while the holes of ZnO will be transferred to the negatively charged Ag_2_O part by the potential energy. Hence, the photoinduced electrons and holes could be effectively separated through charge transfer process at the interface of the two semiconductors, and the photocatalytic process can be described as follows:

(2) $$\mathrm{ZnO}\overset{\mathrm{h\nu}}{\to }h^{+}+e^{-}$$

(3) $${\mathrm{Ag}}_{2}O\overset{\mathrm{h\nu}}{\to }h^{+}+e^{-}$$

(4) $$h^{+}+H_{2}O\overset{\mathrm{h\nu}}{\to }\cdot \mathrm{OH}+H^{+}$$

(5) $$e^{-}+H_{2}O+O_{2}\to \cdot \mathrm{OH}+{\mathrm{OH}}^{\cdot }$$

The results in this paper show that ZnO-Ag_2_O composites have higher photocatalytic activities than pure ZnO and pure Ag_2_O, which is mostly attributed to the inner electric field introduced by the n-type ZnO and p-type Ag_2_O effectively separating the photoinduced electrons and holes.

## Conclusions

Flower-like ZnO-Ag_2_O composites were prepared by a chemical co-precipitating method. The XRD profiles confirm that the composite is composed of cubic-phase Ag_2_O and wurtzite-phase ZnO. Ag_2_O particles decorated on ZnO composite flowers show higher photocatalytic activity than pure components under UV irradiation for the degradation of MO. The activity dependence on the component reveals that the increased Ag_2_O deposited on the composite greatly enhanced the photocatalytic activity, which can be attributed to the p-n junction in the composite effectively inhibiting the recombination of electron–hole pairs.

## Competing interests

The authors declare that they have no competing interests.

## Authors’ contributions

LLX planned the experiments, analyzed the data, and drafted the paper. BW and WLL supervised the project, analyzed the results, and wrote the paper. HLZ performed the experiments and collected the data. CYS and JXC helped with the analysis of the data. All the authors discussed the results and commented on the manuscript. All authors read and approved the final manuscript.

## References

[B1] Fujishima A Honda K Electrochemical photolysis of water at a semiconductor Nature 1972 8 37 38 10.1038/238037a0 12635268

[B2] Hoffmann MR Martin ST Choi W Bahnemann DW Environmental applications of semiconductor photocatalysis Chem Rev 1995 8 69 96 10.1021/cr00033a004

[B3] Duan XW Wang GZ Wang HQ Wang YQ Shen C Cai WP Orientable pore-size-distribution of ZnO nanostructures and their superior photocatalytic activity CrystEngComm 2010 8 2821 2825 10.1039/b922679f

[B4] Zhang L Yang HQ Ma JH Li L Wang XW Zhang LH Tian S Wang XY Controllable synthesis and shape-dependent photocatalytic activity of ZnO nanorods with a cone and different aspect ratios and of short-and-fat ZnO microrods by varying the reaction temperature and time Appl Phys A 2010 8 1061 1067 10.1007/s00339-010-5737-6

[B5] Kowsari E Sonochemically assisted synthesis and application of hollow spheres, hollow prism, and coralline-like ZnO nanophotocatalyst J Nanoparticle Res 2011 8 3363 3376 10.1007/s11051-011-0255-9

[B6] Xu LL Li ZM Cai QH Wang HX Gao H Lu Q Liu J Precursor template synthesis of three-dimensional mesoporous ZnO hierarchical structures and their photocatalytic properties CrystEngComm 2010 8 2166 2172 10.1039/b924097g

[B7] Zhou XF Hu ZL Fan YQ Chen S Ding WP Xu NP Microspheric organization of multilayered ZnO nanosheets with hierarchically porous structures J Phys Chem C 2008 8 11722 11728 10.1021/jp802619j

[B8] Liao DL Badour CA Liao BQ Preparation of nanosized TiO_2_/ZnO composite catalyst and its photocatalytic activity for degradation of methyl orange J Photochem Photobio A: Chem 2008 8 11 19 10.1016/j.jphotochem.2007.07.008

[B9] Lam SM Sin JC Abdullah AZ Mohamed AR Efficient photodegradation of endocrine-disrupting chemicals with Bi_2_O_3_–ZnO nanorods under a compact fluorescent lamp Water Air Soil Pollut 2013 8 1565

[B10] Wu D Jiang Y Yuan Y Wu J Jiang K ZnO–ZnS heterostructures with enhanced optical and photocatalytic properties J Nanoparticle Res 2011 8 2875 2886 10.1007/s11051-010-0176-z

[B11] Wang ZY Huang BB Dai Y Qin XY Zhang XY Wang P Liu HX Yu JX Highly photocatalytic ZnO/In_2_O_3_ heteronanostructures synthesized by a coprecipitation method J Phys Chem C 2009 8 4612 4617 10.1021/jp8107683

[B12] Sapkota BB Mishra SR A simple ball milling method for the preparation of p-CuO/n-ZnO nanocomposite photocatalysts with high photocatalytic activity J Nanosci Nanotechnol 2013 8 6588 6596 10.1166/jnn.2013.7544 24245119

[B13] Chen SF Zhao W Liu W Zhang SJ Preparation, characterization and activity evaluation of p–n junction photocatalyst p-NiO/n-ZnO J Sol-Gel Sci Technol 2009 8 387 396 10.1007/s10971-009-1908-3

[B14] Zhou WJ Liu H Wang J Liu D Du G Cui J Ag_2_O/TiO_2_ nanobelts heterostructure with enhanced ultraviolet and visible photocatalytic activity ACS Appl Mater Interfaces 2010 8 2385 2392 10.1021/am100394x 20735112

[B15] Zhou WJ Liu H Wang J Liu D Du G Han S Lin J Wang R Interface dominated high photocatalytic properties of electrostatic self-assembled Ag_2_O/TiO_2_ heterostructure Phys Chem Chem Phys 2010 8 15119 15123 10.1039/c0cp00734j 20963235

[B16] You Y Wan L Zhang S Xu D Effect of different doping methods on microstructure and photo-catalytic activity of Ag_2_O–TiO_2_ nanofibers Mater Res Bull 2010 8 1850 1854 10.1016/j.materresbull.2010.09.015

[B17] Zhu L Wei B Xu LL Lu Z Zhang HL Gao H Che JX Ag_2_O-Bi_2_O_3_ composites: synthesis, characterization and high efficient photocatalytic activities CrystEngComm 2012 8 5705 5709 10.1039/c2ce25301a

[B18] Ansari SA Khan MM Kalathil S Nisar A Lee J Cho MH Oxygen vacancy induced band gap narrowing of ZnO nanostructures by an electrochemically active biofilm Nanoscale 2013 8 9238 9246 10.1039/c3nr02678g 23938937

[B19] Wu X Li WK Wang H Facile fabrication of porous ZnO microspheres by thermal treatment of ZnS microspheres J Hazard Mater 2010 8 573 580 10.1016/j.jhazmat.2009.09.090 19913355

[B20] Jing LQ Yuan FL Hou HJ Xin BF Cai WM Fu HG Relationships of surface oxygen vacancies with photoluminescence and photocatalytic performance of ZnO nanoparticles Sci Chi Series B: Chem 2005 8 25 30

[B21] Xu J Chang YG Zhang YY Ma SY Qu Y Xu CT Effect of silver ions on the structure of ZnO and photocatalytic performance of Ag/ZnO composites Appl Surf Sci 2008 8 1996 1999 10.1016/j.apsusc.2008.06.130

[B22] Duan L Lin B Zhang W Zhong S Enhancement of ultraviolet emissions from ZnO films by Ag doping Appl Phys Lett 2006 8 232110 10.1063/1.2211053

[B23] Niu BJ Wu LL Tang W Zhang XT Meng QG Enhancement of near-band edge emission of Au/ZnO composite nanobelts by surface plasmon resonance CrystEngComm 2011 8 3678 3681 10.1039/c1ce05175j

[B24] Lin XP Xing JC Wang WD Shan ZC Xu FF Huang FQ Photocatalytic activities of heterojunction semiconductors Bi_2_O_3_/BaTiO_3_: a strategy for the design of efficient combined photocatalysts J Phys Chem C 2007 8 18288 18293 10.1021/jp073955d

